# Influence of China’s 2009 healthcare reform on the utilisation of continuum of care for maternal health services: evidence from two cross-sectional household surveys in Shaanxi Province

**DOI:** 10.1186/s12939-020-01179-3

**Published:** 2020-06-19

**Authors:** Xiaojing Fan, Meghan Bruce Kumar, Zhongliang Zhou, Ching-Hung Lee, Duolao Wang, Haixia Liu, Shaonong Dang, Jianmin Gao

**Affiliations:** 1 School of Public Policy and Administration, Xi’an Jiaotong University, No. 28 Xianning West Road, Xi’an, 710049 Shaanxi China; 2 Department of International Public Health, Liverpool School of Tropical Medicine, L3 5QA, Liverpool, UK; 3 MARCH Centre, London School of Hygiene and Tropical Medicine, London, WC1E 7HT UK; 4 Department of Clinical Sciences, Liverpool School of Tropical Medicine, Liverpool, L3 5QA UK; 5 School of Public Health and Management, Binzhou Medical University, Shandong, Yantai, 264003 China; 6 Department of Epidemiology and Health Statistics, School of Public Health, Center of Medical Science, Xi’an Jiaotong University Health Science Center, 76 Yanta West Road, Xi’an, 710061 China

**Keywords:** Healthcare reform, Continuum of care for maternal health services, Equity, National Health Service Surveys, China

## Abstract

**Background:**

Continuum of care for maternal health services (CMHS) is a proven approach to improve health and safety for mothers and newborns. This study aims to explore the influence of China’s 2009 healthcare reform on improving the CMHS utilisation.

**Methods:**

This population-based cross-sectional quantitative study included 2332 women drawn from the fourth and fifth National Health Service Surveys of Shaanxi Province, conducted in 2008 and 2013 respectively, before and after China’s 2009 healthcare reform. A generalised linear mixed model (GLMM) was applied to analyse the influence of this healthcare reform on utilisation of CMHS. Concentration curves, concentration indexes and its decomposition method were used to analyse the equity of changes in utilisation.

**Results:**

This study showed post-reform CMHS utilisation was higher in both rural and urban women than the CMHS utilisation pre-reform (according to China’s policy defining CMHS). The rate of CMHS utilisation increased from 24.66 to 41.55% for urban women and from 18.31 to 50.49% for rural women (urban: χ^2^ = 20.64, *P* < 0.001; rural: χ^2^ = 131.38, P < 0.001). This finding is consistent when the WHO’s definition of CMHS is applied for rural women after reform (12.13% vs 19.26%; χ^2^ = 10.99, *P* = 0.001); for urban women, CMHS utilisation increased from 15.70 to 20.56% (χ^2^ = 2.57, *P* = 0.109). The GLMM showed that the rate of CMHS utilisation for urban women post-reform was five times higher than pre-reform rates (OR = 5.02, 95%CL: 1.90, 13.31); it was close to 15 times higher for rural women (OR = 14.70, 95%CL: 5.43, 39.76). The concentration index for urban women decreased from 0.130 pre-reform (95%CI: − 0.026, 0.411) to − 0.041 post-reform (95%CI: − 0.096, 0.007); it decreased from 0.104 (95%CI: − 0.012, 0.222) to 0.019 (95%CI: − 0.014, 0.060) for rural women. The horizontal inequity index for both groups of women also decreased (0.136 to − 0.047 urban and 0.111 to 0.019 for rural).

**Conclusions:**

China’s 2009 healthcare reform has positively influenced utilisation rates and equity of CMHS’s utilisation among both urban and rural women in Shaanxi Province. Addressing economic and educational attainment gaps between the rich and the poor may be effective ways to improve the persistent health inequities for rural women.

## Introduction

The maternal mortality ratio (MMR) has rapidly reduced globally during the past decades, declining from 385 to 216 maternal deaths per 100,000 livebirths between 1990 and 2015 [1]. Despite global progress in reducing maternal mortality, MMR in low- and middle-income countries (LMICs) is still seven times higher than in high-income countries [2]. In China, the current MMR of 27 per 100,000 live births is still 2–6 times that of developed countries (e.g. 14 deaths per 100,000 in USA; 9 deaths per 100,000 in United Kingdom; 4 deaths per 100,000 in Italy and Sweden), as described by the World Health Organisation (WHO) [2]. Prenatal care penetration is high, with more than 80% of pregnant women attending ≥4 antenatal visits and delivering in hospital, yet only 25% of women receive ≥3 postnatal visits within 42 days after delivery [3, 4]. One possible means of further reducing the MMR in China would be improving adherence to postnatal visits through continuity of care (COC). COC requires access to care throughout one’s lifecycle, including adolescence, pregnancy, childbirth, the postnatal period, and childhood [5]. Scholars have proposed COC as a key framework for tracking maternal and neonatal health and assessing reductions in maternal and neonatal deaths [5, 6]. COC for maternal health services (CMHS) utilisation is defined by pregnant women attending antenatal visits, delivery in health facilities and receiving postnatal visits from health professionals in their homes continuously from pregnancy to 42 days after delivery [7]. Studies from Lancet and PLOS ONE showed adherence to full CMHS can reduce neonatal mortality by 36–67% and reduce combined perinatal and maternal mortality by 15% [8, 9]. However, CMHS has not been adequately implemented and assessed in low- and middle-income countries (LMICs) [10–12].

China embarked on a comprehensive healthcare reform in 2009 aimed at providing all citizens with equal access to basic health care with reasonable quality and sufficient financial risk protection [13]. The reform established the national primary and critical public health service in order to promote health for all, with a focus on attending prenatal visits, hospital delivery and receiving postnatal visits at the full maternal period [14–16]. In China, existing research on the healthcare reform impacts primarily focuses on prenatal visits, hospital delivery or postnatal visits separately. Recent studies have evaluated socio-economic inequalities and found socioeconomic determinants of prenatal and postnatal visits [17–19], explored influence of health policy on improving the utilisation of hospital delivery [20], prenatal or postnatal visits [21], fewer studies explore the utilisation of CMHS. To date, there has been some research exploring the determinants, effects, value and measurement of CMHS in LMICs outside of China: in Lao PDR [22], Nepal [23], Tanzania [10], South Africa [11], Cambodia [24]. This study aimed to fill the study gap on exploring the influence of health policy on improving the utilisation of CMHS in one province of China. We hypothesise that the utilisation of CMHS has improved after the introduction of the comprehensive health care reform.

In this study, two rounds of a representative cross-sectional household survey are used to analyse the influence of the 2009 Chinese healthcare reform on the utilisation of CMHS in Shaanxi Province. This study also provides policy recommendations for further improving maternal health care utilisation in China and narrowing the gap in MMR between LMICs and high-income countries.

## Methods

### Study design and sample

This study analyses the influence of the 2009 Chinese healthcare reform on CMHS utilisation in Shaanxi Province. Shaanxi Province, in the west of China, was selected as the study area because it is the type of region that the reform intended to target: predominantly rural (48.69% of population) and having a high proportion of low socioeconomic status in the population. By the end of 2013, Shaanxi had a population of roughly 37.60 million with a per capital Gross Regional Product (GRP) of 42,692 Chinese yuan. In the same year, the birth rate was 10.01% and the natural growth rate was 3.86% [25].

The National Health Service Survey (NHSS) is a population-based cross-sectional nationally representative survey commissioned by the China’s National Health Commission every 5 years [26–28]. Based on the structure of Chinese administrative districts and the imbalanced population distributions among the different provinces, a multi-stage stratified cluster randomized design was used to provide a representative sample in each province. Data presented in this paper were drawn from the fourth (in 2008) and fifth (in 2013) NHSS conducted in Shaanxi province before and after the 2009 Chinese healthcare reform.

In each survey round, face-to-face interviews were collected by the investigators trained by China’s National Health Commission using a household health questionnaire that mainly included open-ended questions (see Supplementary Questionnaire S1 and S2 online). Data on maternal socio-economic status (including area, age, education, health insurance, annual personal expenditure, employment) as well as chronic disease, parity, antenatal visits, hospital delivery and postnatal visits from pregnancy to 42 days after delivery were collected in the interview. During data collection, experts provided supervision and revisited 5% of the sampled households to check the accuracy of data recorded by interviewers. They asked 14 key questions again to check the consistency of the information recorded and the consistency should be at least 95%. The Myer’s Blended Index was used to assess the representativeness of the sample (1.67 in the 4th NHSS and 1.62 in 5th NHSS), indicating that in both surveys there was no significant difference between the sampled age distribution and the overall age distribution of Shaanxi Province [21, 29].

**Fig. 1:**
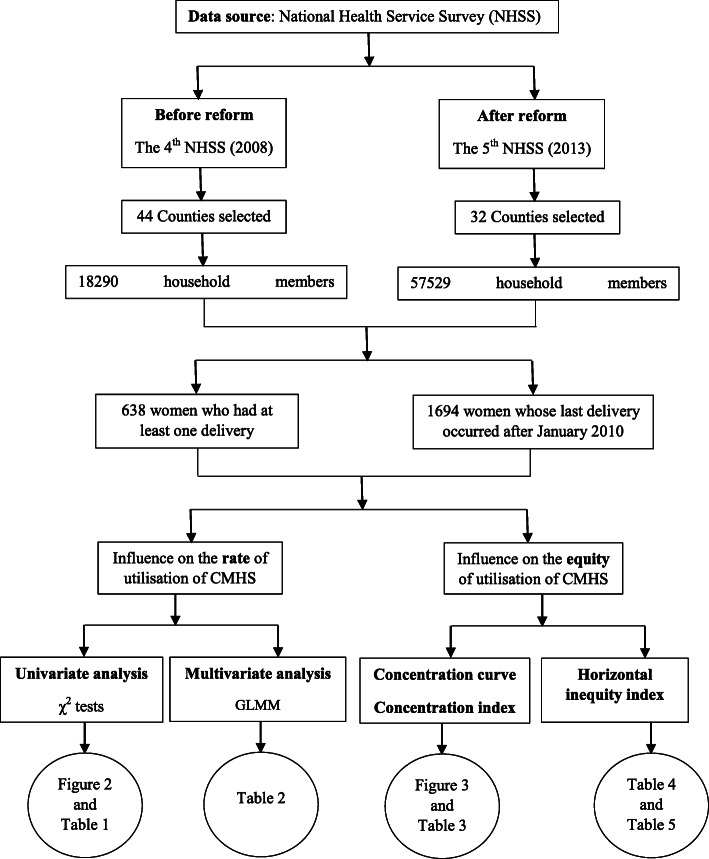
Study flow chart

Details of the NHSS sampling and data that are included in this paper are provided in Fig. 1. Specifically, we only included women whose last delivery occurred after January 2010, considering the official inception date of the health system reform (September 2009). This gave us a sample of 638 women in the fourth NHSS and 1694 women in the fifth NHSS in this analysis.

### Indicators

China’s 2009 healthcare reform in this study refers to a series of measures introduced and implemented after 2009 to strengthen women’s maternal health care, mainly including through the national public health service. According to WHO definition, the utilisation of CMHS is categorized as: women who attended ≥4 prenatal visits, had hospital delivery and received ≥3 postnatal visits from pregnancy to 42 days after delivery [30]. In the level of China, the utilisation of CMHS is categorized as: women who attended ≥5 prenatal visits, had hospital delivery and received ≥1 postnatal visit(s) from pregnancy to 42 days after delivery [31, 32]. We analysed rural and urban population separately and compared them to look at geographic equity, allowing us to account for the difference between rural and urban populations in terms of income, education and health service utilisation [33].

### Statistical analysis

In this study, sample data has been checked for missing data and outliers and cleaned prior to data analysis. Descriptive analysis was performed to show the demographic information of maternal women in the sample and their CMHS status. A generalised linear mixed model (GLMM) including both fixed and random effects was used in this study to show the association between China’s 2009 healthcare reform and CMHS utilisation when controlling for other confounding factors. The healthcare reform was specified as fixed effects, women’s family code as a random effect; maternal women’s age, education, employment, annual personal expenditure, health score, health insurance, chronic disease and parity were included as covariates. The model we used was as following:

1 $$ \eta \left({y}_{ij}\right)={\beta}_{0j}+{\beta}_{1j}{x}_{1i}+{\beta}_{2j}{x}_{2i}+\cdots +{\beta}_{pj}{x}_{pi}+{\varepsilon}_{ij} $$

In Eq. (1), the linear prediction η is the combination of the fixed and random effects excluding the residuals. *y*_*ij*_ is the rate of CMHS utilisation. *β*_0*j*_ is a constant, *β*_*pj*_ represents the effects of *x*_*pi*_ on *y*, and *ε*_*ij*_ is a random error. The link function is binomial.

Concentration curve, concentration index (CI) and horizontal inequity index (HI) were used to measure the equity of CMHS utilisation. Before measuring equity, we first measured inequality. Concentration curve and CI were used to measure the extent of income-related inequality of CMHS utilisation. This is calculated as twice the area between the concentration curve and the line of equality and changed from − 1 to 1 [34]. A positive concentration index means that high-income women utilize more CMHS utilisation than their low-income counterparts and negative one means the low-income group utilizes more CMHS utilisation than their rich counterparts, the formula is as following:

2 $$ C=\frac{2}{\mu }{\mathit{\operatorname{cov}}}_w\left({\mathrm{y}}_i,{R}_i\right) $$

where *C* stands for concentration index, *y*_*i*_ is CMHS utilisation index, *μ* is the mean of CMHS utilisation index, and *R*_*i*_ is the fractional rank of annual personal consumption expenditure distribution.

Inequality can be further explained by decomposing the concentration index into its determining components, then horizontal inequity index (HI) can be computed by subtracting the contribution of need variables (such as women’s age, health score and chronic disease) from the concentration index of CMHS utilisation; it is a summary measure of the magnitude of inequity in the dependent variable [35]. These determinants were selected according to previous research but constrained by the variables collected in the investigation [22, 36]. A probit regression model was used to indirectly standardize the CMHS utilisation since the outcome variable is binary. As the standardization of health utilisation holds for a linear model of healthcare, we applied the linear approximation to the probit model to extract marginal effects of each determinant on observed probabilities of the outcome variable. The formula for the concentration index decomposition can be written as follows:

3 $$ {y}_i=G\left(\alpha +{\sum}_j{\beta}_j{x}_{ji}+{\sum}_k{\gamma}_k{z}_{ki}\right)+{\varepsilon}_i $$

*G* is functional transformation, *y* is the dependent variable, *x*_*ji*_ are needs variables, and *z*_*ki*_ are control variables. Then the standardized need was estimated using the following equation:

4 $$ {\hat{y}}_i^{IS}={y}_i-G\left(\hat{\alpha}+{\sum}_j{\hat{\beta}}_j{x}_{ji}+{\sum}_k{\hat{\gamma}}_k{\overline{z}}_k\right)+\frac{1}{n}\times {\sum}_{i=1}^nG\left(\hat{\alpha}+{\sum}_j{\hat{\beta}}_j{x}_{ji}+{\sum}_k{\hat{\gamma}}_k{\overline{z}}_k\right) $$

where ${\hat{y}}_i^{IS}$ is standardized continuum of maternal health service utilisation, *n* is sample size. The more CMHS allocated to the population with greater need, the less inequity of CMHS utilisation.

The statistical analyses were performed using STATA statistical software version 12.0 (StataCorp LP, College station 77,845, USA). A two-tailed *P* value < 0.05 was considered statistically significant.

## Results

### Increases in rate of utilisation of CMHS post-reform

**Fig. 2:**
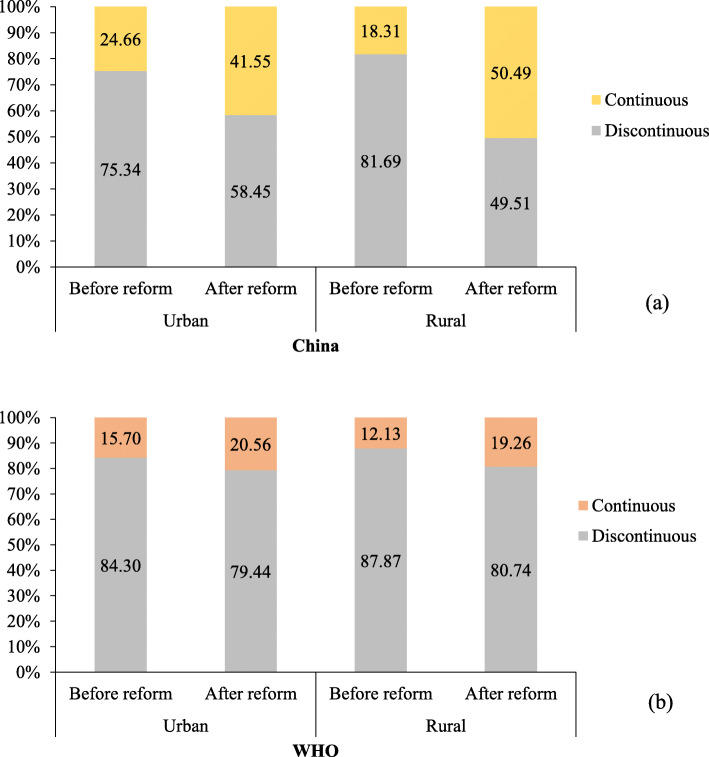
Comparison of continuous maternal health service utilisation before and after healthcare reform

According to China’s definition of CMHS, there were increases in utilisation rate after China’s 2009 healthcare reform both for urban and rural women (urban: *χ*^*2*^ = 20.64, *P* < 0.001; rural: *χ*^*2*^ = 131.38, *P* < 0.001; Table 1) compared with the rate of CMHS utilisation pre-reform. This finding is consistent when the WHO definition of CMHS is applied for rural women only (12.13% vs 19.26%; *χ*^*2*^ = 10.99, *P* = 0.001), as shown in Fig. 2. For urban women, the rate of CMHS utilisation also increased under the WHO criteria from 15.70 to 20.56%, but this was not a significant change (*χ*^*2*^ = 2.57, *P* = 0.109).

**Table 1:** Distribution of continuum of care for maternal health service utilisation among maternal women’s socioeconomic characteristics (*n* = 2332)

Variables	Urban (*n* = 912)			Rural (*n* = 1420)		
	No	Yes	*P*	No	Yes	*P*
Healthcare reform			< 0.001			< 0.001
Before	167(29.61)	55(15.80)		336(40.88)	80(13.38)	
After	397(70.39)	293(84.20)		486(59.12)	518(86.62)	
Age (years)			0.019			0.789
≤ 25	142(25.18)	104(29.89)		278(33.82)	210(35.12)	
26–30	239(42.38)	161(46.26)		258(31.39)	190(31.77)	
≥ 31	183(32.45)	83(23.85)		286(34.79)	198(33.11)	
Education			0.025			< 0.001
≤ Primary school	62(10.99)	20(5.78)		250(30.53)	98(16.39)	
Middle school	272(48.23)	171(49.42)		460(56.17)	381(63.71)	
≥ High school	230(40.78)	155(44.80)		109(13.31)	119(19.90)	
Employment			0.090			0.885
No	206(36.52)	108(31.03)		126(15.33)	90(15.05)	
Yes	358(63.48)	240(68.97)		696(84.67)	508(84.95)	
Annual personal expenditure (Chinese Yuan)			0.314			< 0.001
Poorest	60(10.66)	30(8.62)		270(32.89)	104(17.42)	
Poorer	86(15.28)	53(15.23)		192(23.39)	132(22.11)	
Middle	120(21.31)	63(18.10)		144(17.54)	142(23.79)	
Richer	114(20.25)	89(25.57)		140(17.05)	123(20.60)	
Richest	183(32.50)	113(32.47)		75(9.14)	96(16.08)	
Health score	86.54 ± 8.78	88.58 ± 7.61	< 0.001	85.93 ± 9.20	87.45 ± 8.39	< 0.001
Health insurance			0.033			0.280
No	92(16.31)	39(11.21)		24(2.92)	12(2.01)	
Yes	472(83.69)	309(88.79)		798(97.08)	586(97.99)	
Chronic disease			0.024			0.510
No	536(95.04)	341(97.99)		792(96.35)	580(96.99)	
Yes	28(4.96)	7(2.01)		30(3.65)	18(3.01)	
Parity			0.064			0.015
1	368(65.36)	248(71.26)		402(49.14)	332(55.70)	
≥ 2	195(34.64)	100(28.74)		416(50.86)	264(44.30)

In specific, the GLMM shows that the rates of the utilisation of CMHS after China’s 2009 healthcare reform were nearly 5 times (OR = 5.02, 95%CL:1.90,13.31) higher for urban women and 15 times (OR = 14.70, 95%CL:5.43,39.76) higher for rural women than the rates before healthcare reform (after adjusting for maternal age, education, employment, annual personal expenditure, health score, health insurance, chronic disease and parity, Table 2). Urban and rural women with higher education and health insurance had higher rates of CMHS utilisation after adjusting for other characteristics (*P* < 0.05; Table 2). In addition, rural women over 31 years old (OR = 2.49, 95%CL: 1.23, 5.06) and primigravida (OR = 0.40, 95%CL: 0.21, 0.75) had higher rates of utilisation.

**Table 2:** Determinants of continuum of care for maternal health service utilisation by generalised linear mixed model (*n* = 2332)

Variables	Urban (*n* = 912)			Rural (n = 1420)		
	OR	95%CL	*P*	OR	95%CL	*P*
Healthcare reform						
Before	1.00			1.00		
After	5.02	1.90,13.31	0.001	14.70	5.43,39.76	< 0.001
Age (years)						
≤ 25	1.00			1.00		
26–30	1.10	0.62,1.96	0.741	1.40	0.80,2.44	0.239
≥ 31	0.78	0.38,1.59	0.493	2.49	1.23,5.06	0.012
Education						
≤ Primary school	1.00			1.00		
Middle school	2.62	0.98,7.03	0.056	3.11	1.64,5.92	0.001
≥ High school	4.18	1.30,13.44	0.016	4.55	1.88,11.01	0.001
Employment						
No	1.00			1.00		
Yes	1.47	0.86,2.49	0.156	1.12	0.62,2.03	0.697
Annual personal expenditure (Chinese Yuan)						
Poorest	1.00			1.00		
Poorer	0.94	0.35,2.50	0.899	1.27	0.67,2.41	0.454
Middle	0.64	0.24,1.67	0.357	1.70	0.85,3.41	0.132
Richer	0.96	0.37,2.47	0.934	1.04	0.51,2.12	0.910
Richest	0.51	0.19,1.37	0.183	1.94	0.86,4.39	0.112
Health score	1.03	1.00,1.07	0.051	1.02	0.99,1.05	0.147
Health insurance						
No	1.00			1.00		
Yes	2.67	1.13,6.30	0.026	4.25	1.00,18.13	0.05
Chronic disease						
No	1.00			1.00		
Yes	0.35	0.08,1.49	0.156	0.75	0.23,2.50	0.642
Parity						
1	1.00			1.00		
≥ 2	0.62	0.32,1.20	0.155	0.40	0.21,0.75	0.005

Abbreviations: *OR* odds ratio, *CL* confidence limits

### Improvement in equity of utilisation of CMHS post-reform

**Fig. 3:**
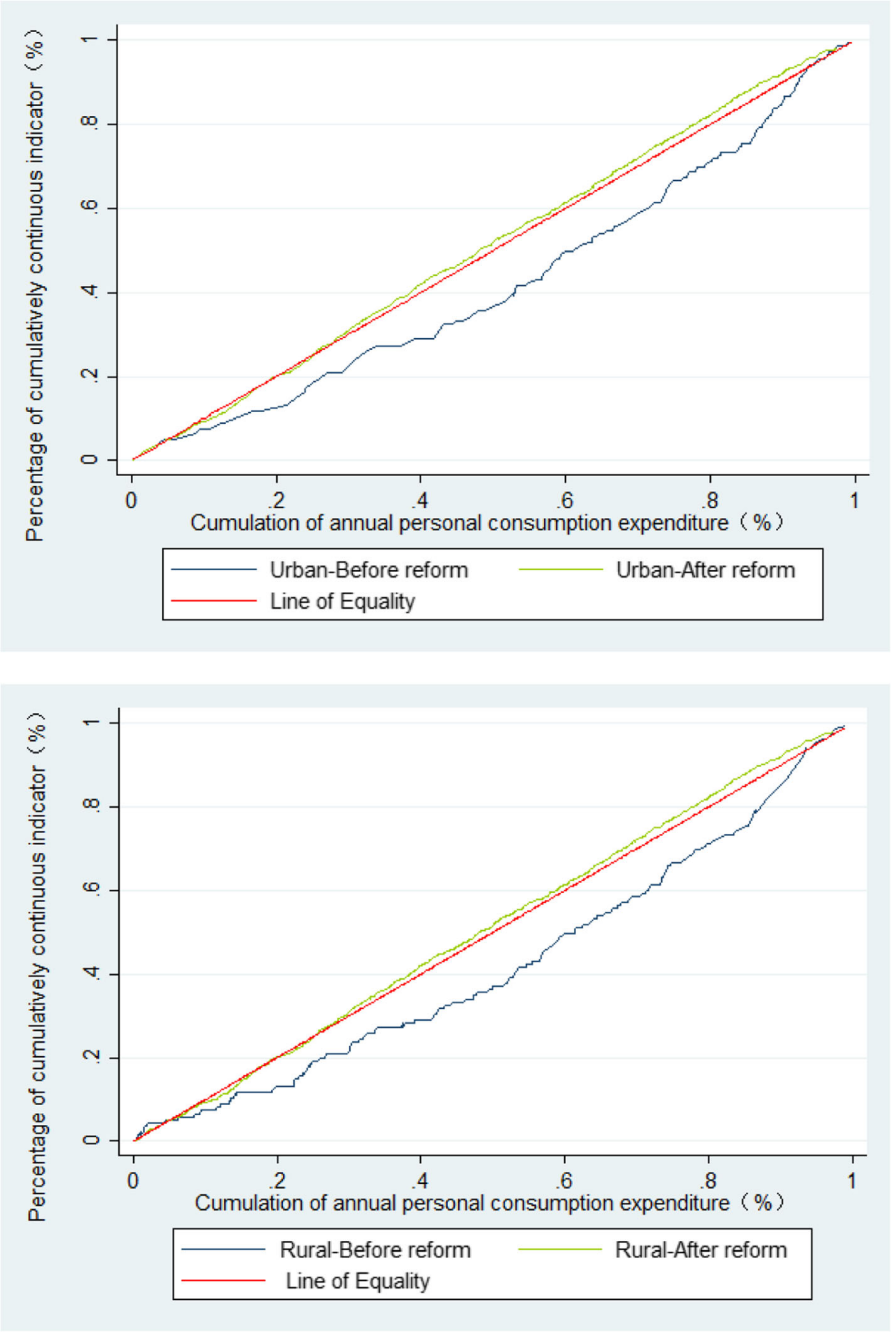
Concentration curves on continuum of care for maternal health service utilisation before and after healthcare reform

Figure 3 shows that before the 2009 reform, concentration curves both in urban and rural women lay significantly below the line of equality, indicating that the utilisation of CMHS was more concentrated among the rich. However, the concentration curves lay above the line of equality after reform. In addition, the CI of occurring CMHS utilisation in urban women decreased significantly (*P* = 0.021) from 0.130 (95% CL: − 0.026, 0.411) to − 0.041 (95% CL: − 0.096, 0.007). This decreasing trend is also shown for rural women but still favors the rich and is not statistically significant (*P* = 0.170): specifically, the CI among rural women was 0.104 (95% CL: − 0.012, 0.222) before reform, and after 0.019 (95% CL: − 0.014, 0.060, Table 3).

**Table 3:** Inequality of urban and rural women’s continuum of care for maternal health service utilisation before and after healthcare reform (*n* = 2332)

Area	Before healthcare reform			After healthcare reform			*P*
	CI	95% CL		CI	95% CL		
		Lower	Upper		Lower	Upper	
Urban	0.130	−0.026	0.411	− 0.041	− 0.096	0.007	0.021
Rural	0.104	−0.012	0.222	0.019	− 0.014	0.060	0.170

Abbreviations: *CI* concentration index, *CL* confidence limits

The majority of the CMHS inequality was attributable to education, economic statuses and health insurance by defining the contributions as a proportion of each variable; Tables 4 and 5 present the decomposition of CIs of CMHS utilisation, describing the contribution of different population variables to the inequality of CMHS utilisation and the proportion of contribution in the overall CIs. A positive (negative) contribution represented the variable raised (reduced) the pro-rich inequality. For CMHS utilisation among urban women, we found that economic status, health insurance and parity had the largest (112.36%), second-largest (24.08%) and third-largest (11.99%) contributions respectively to the inequality of CMHS. For rural women, economic status, education and health insurance had the largest (52.12%), second-largest (38.53%) and third-largest (10.01%) contributions respectively to the inequality of CMHS utilisation. HI of CMHS utilisation post-reform was − 0.047 for urban women, evidencing a pro-poor inequity; the horizontal inequity index was 0.019 for rural women and indicating a pro-rich inequity (Tables 4 and 5).

**Table 4:** Decomposition analysis of concentration index on urban women’s continuum of care for maternal health service utilisation before and after healthcare reform (*n* = 912)

Variables	Before healthcare reform (*n* = 222)				After healthcare reform (*n* = 690)			
	Elasticity	CI	Contribution to CI	%	Elasticity	CI	Contribution to CI	%
Age (years)								
≤ 25	Reference				Reference			
26–30	− 0.286	0.062	− 0.018	−13.70	0.080	0.033	0.002	−6.42
≥ 31	−0.138	0.012	−0.002	−1.30	0.005	−0.039	− 0.0002	0.50
Education								
≤ Primary school	Reference				Reference			
Middle school	1.491	−0.234	−0.350	− 268.90	0.200	− 0.077	− 0.015	37.40
≥ High school	2.210	0.175	0.387	297.50	0.135	0.142	0.019	−46.98
Employment								
No	Reference				Reference			
Yes	−0.029	−0.444	0.001	0.99	0.153	−0.009	−0.001	3.37
Annual personal expenditure (Chinese Yuan)								
Poor	Reference				Reference			
Poorer	0.055	−0.069	−0.004	−2.96	− 0.056	−0.770	0.043	− 105.54
Middle	−0.004	0.280	−0.001	−0.96	− 0.104	−0.357	0.037	−90.68
Richer	0.072	0.449	0.033	25.01	−0.107	−0.066	− 0.007	17.16
Richest	0.040	0.545	0.022	16.95	−0.253	0.480	−0.121	296.16
Health score	2.961	0.005	0.014	10.47	0.621	0.003	0.002	−4.74
Health insurance								
No	Reference				Reference			
Yes	0.289	−0.078	−0.023	−17.40	0.275	−0.036	− 0.010	24.08
Chronic disease								
No	Reference				Reference			
Yes	−0.012	0.029	− 0.0003	−0.27	− 0.012	−0.202	0.002	−6.04
Parity								
1	Reference				Reference			
≥ 2	−0.219	−0.029	0.006	4.82	−0.281	−0.018	0.005	−11.99
Needs variables	–	–	−0.006	−4.80	–	–	0.006	−16.70
HI^4^	–	–	0.136	–	–	–	−0.047	–

Abbreviations: *CI* concentration index, *%* pure percentage contributions of determinants to the socioeconomic inequality in continuum of maternal health service utilisation, *HI* horizontal inequity index. Needs variables mean contribution of factors to *CI*, including age, health score and chronic disease

**Table 5:** Decomposition analysis of concentration index on rural women’s continuum of care for maternal health service utilisation before and after healthcare reform (*n* = 1420)

Variables	Before healthcare reform (*n* = 416)				After healthcare reform (*n* = 1004)			
	Elasticity	CI	Contribution to CI	%	Elasticity	CI	Contribution to CI	%
Age (years)								
≤ 25	Reference				Reference			
26–30	−0.004	0.033	−0.0001	−0.14	0.031	−0.014	− 0.0004	−2.29
≥ 31	0.284	−0.023	−0.007	−6.38	0.058	−0.022	− 0.001	− 6.69
Education								
≤ Primary school	Reference				Reference			
Middle school	0.350	0.029	0.010	9.70	0.160	−0.011	−0.002	−8.87
≥ High school	0.135	0.131	0.018	17.09	0.058	0.154	0.009	47.40
Employment								
No	Reference				Reference			
Yes	−0.104	0.0003	−0.0001	− 0.03	0.059	0.002	0.0001	0.64
Annual personal expenditure (Chinese Yuan)								
Poor	Reference				Reference			
Poorer	0.048	0.288	0.014	13.29	0.005	−0.413	−0.002	−11.71
Middle	0.032	0.661	0.021	20.16	0.025	−0.008	− 0.002	−10.57
Richer	0.006	0.875	0.006	5.316	−0.005	0.329	−0.002	−9.12
Richest	0.034	1.072	0.036	34.82	0.019	0.755	0.015	77.19
Health score	−0.005	−0.001	0.000	0.004	0.620	0.002	0.001	6.33
Health insurance								
No	Reference				Reference			
Yes	0.722	0.0004	0.0003	0.29	0.303	−0.006	−0.002	−10.01
Parity								
1	Reference				Reference			
≥2	−0.918	−0.005	0.004	4.22	−0.315	−0.003	0.001	4.38
Needs variables	–	–	−0.007	−6.52	–	–	−0.0004	−2.65
HI^4^	–	–	0.111	–	–	–	0.0194	–

Abbreviations: *CI* concentration index; *%* Pure percentage contributions of determinants to the socioeconomic inequality in continuum of maternal health service utilisation; *HI* Horizontal inequity index. Needs variables mean contribution of factors to *CI*, including age and health score

## Discussion

CMHS is one of the ways to improve maternal health and should be effective both in policy and in reality [37, 38]. This is the first known study to measure the influence of China’s 2009 healthcare reform on the utilisation of CMHS in Shaanxi Province. In the 10 years since China’s 2009 healthcare reform, many studies focused on different geographies and health conditions have demonstrated its contribution to improving population health status [15, 39–41]. In this study, we found the 2009 healthcare reform has had a positive influence on improving the rate and equity of CMHS utilisation for both urban and rural women. The horizontal inequity index of CMHS utilisation decreased from 0.111 to 0.019 among rural women after the healthcare reform, but remains more concentrated among the richer rural women. Findings from the decomposition of inequality in rural CMHS indicated the horizontal inequity index were mainly explained by educational and economic status (annual personal expenditure), conforming with previous studies analysing health inequity [26, 27]. The educational and economic status were positively associated with CMHS and have positive contribution, highlighting that the richer and more educated rural women were more likely to have CMHS. One potential reason to explain this was that the richer and more educated individuals could be better accessible to healthcare [18, 19]. Therefore, the contributions of key determinants should be considered by policy-makers when formulating health policy interventions.

According to the concept and principles of maternal health services, to achieve full health for women throughout the pregnancy period, each woman should have ≥4 times antenatal visits, skilled delivery and ≥ 3 times postnatal visits throughout the maternity period [42], with CMHS putatively improving self-awareness and utilisation rates. However, this study found that the utilisation rates of CMHS have increased after China’s 2009 healthcare reform but still remains low. Studies in other LMICs have shown that the rate of CMHS utilisation is low because of shortages in human, financial resources and inadequate health-system infrastructure (although they used a different way to assess the continuum of care). Studies showed 6.8% of maternal women completed the continuum of maternal, newborn and child health services in a rural district of Lao People’s Democratic Republic [22]; 5.0% of maternal women completed at least four antenatal visits, hospital delivery and at least once postnatal visits continuously in Ratanakiri, Cambodia [12]; 41% of maternal women completed at least one antenatal visit, hospital delivery and at least one postnatal visit continuously in Nepal [23]; 7.9% of women completed the continuum of care through continuous visits to health facilities in Ghana [43]. The post-reform survey data in this study showed only 20.56% of urban women and 19.26% of rural women made CMHS. Exploring the determinants of the CMHS’s utilisation rate, education and health insurance were positively associated with this rate. Considering the poor maternal health outcomes achieved in China, more efforts should be made for policy-makers to increase women’s education and coverage health insurance in order to improve the rate of continuum of care for maternal health service.

### Limitations

This is the first study, to our knowledge, that studied the influence of China’s 2009 healthcare reform on the CMHS utilisation in Shaanxi Province. However, there are some limitations to this study. Firstly, all the data were self-reported and therefore may include recall or social desirability bias. However, the recall bias is likely to be small because pregnancy and childbirth are events that women remember for years [18]. Secondly, the measured determinants of CMHS utilisation available are limited by the pre-specified questions in the survey and there could be some potential unobserved confounding factors for which we did not control. Lastly, the imbalanced sample size before (*n* = 638) and after (*n* = 1694) the reform of interest may have some potential impacts on the results and conclusions, such as potentially introducing more selection bias and resulting with larger standard error and reduced statistical significance.

## Conclusions

This study showed China’s 2009 healthcare reform has had positive influence on improving the rate and equity of CMHS utilisation for both urban and rural women in Shaanxi province. Addressing economic and educational gaps between the rich and the poor should be considered by policy-makers when formulating health policy interventions to improve health inequities for rural women.

## Supplementary information

**Additional file 1: Supplementary Questionnaire S1.** The household questionnaire of the fourth National Health Service Survey. **Supplementary Questionnaire S2.** The household questionnaire of the fifth National Health Service Survey.

## Data Availability

This data was drawn from the fourth and fifth National Health Services Survey of Shaanxi Province. They are available from the Shaanxi National Health Commission for researchers who meet the criteria for access to confidential data, and are not opened to everyone. Researchers who want to use these data should contact Zhongliang Zhou (zzliang1981@163.com).

## Abbreviations

CMHS: Continuum of care for maternal health services
CI: Concentration Index
CL: Confidence Limits
LMICs: Low and Middle Income Countries
MMR: Maternal mortality rate
NHSS: National Health Service Survey
OR: Odds risk
WHO: World Health Organisation
